# Structural and Mechanistic Insights Into Dimethylsulfoxide Formation Through Dimethylsulfide Oxidation

**DOI:** 10.3389/fmicb.2021.735793

**Published:** 2021-09-24

**Authors:** Xiu-Juan Wang, Nan Zhang, Zhao-Jie Teng, Peng Wang, Wei-Peng Zhang, Xiu-Lan Chen, Yu-Zhong Zhang, Yin Chen, Hui-Hui Fu, Chun-Yang Li

**Affiliations:** ^1^Frontiers Science Center for Deep Ocean Multispheres and Earth System, College of Marine Life Sciences, Ocean University of China, Qingdao, China; ^2^State Key Laboratory of Microbial Technology, Marine Biotechnology Research Center, Shandong University, Qingdao, China; ^3^Laboratory for Marine Biology and Biotechnology, Pilot National Laboratory for Marine Science and Technology, Qingdao, China; ^4^School of Bioengineering, Qilu University of Technology, Jinan, China; ^5^School of Life Sciences, University of Warwick, Coventry, United Kingdom

**Keywords:** DMS, DMSO, flavin-containing monooxygenase, SAR11, catalytic mechanism

## Abstract

Dimethylsulfide (DMS) and dimethylsulfoxide (DMSO) are widespread in marine environment, and are important participants in the global sulfur cycle. Microbiol oxidation of DMS to DMSO represents a major sink of DMS in marine surface waters. The SAR11 clade and the marine *Roseobacter* clade (MRC) are the most abundant heterotrophic bacteria in the ocean surface seawater. It has been reported that trimethylamine monooxygenase (Tmm, EC 1.14.13.148) from both MRC and SAR11 bacteria likely oxidizes DMS to generate DMSO. However, the structural basis of DMS oxidation has not been explained. Here, we characterized a Tmm homolog from the SAR11 bacterium *Pelagibacter* sp. HTCC7211 (Tmm_7211_). Tmm_7211_ exhibits DMS oxidation activity *in vitro*. We further solved the crystal structures of Tmm_7211_ and Tmm_7211_ soaked with DMS, and proposed the catalytic mechanism of Tmm_7211_, which comprises a reductive half-reaction and an oxidative half-reaction. FAD and NADPH molecules are essential for the catalysis of Tmm_7211_. In the reductive half-reaction, FAD is reduced by NADPH. In the oxidative half-reaction, the reduced FAD reacts with O_2_ to form the C4a-(hydro)peroxyflavin. The binding of DMS may repel the nicotinamide ring of NADP^+^, and make NADP^+^ generate a conformational change, shutting off the substrate entrance and exposing the active C4a-(hydro)peroxyflavin to DMS to complete the oxidation of DMS. The proposed catalytic mechanism of Tmm_7211_ may be widely adopted by MRC and SAR11 bacteria. This study provides important insight into the conversion of DMS into DMSO in marine bacteria, leading to a better understanding of the global sulfur cycle.

## Introduction

Dimethylsulfide (DMS), one of the major biogenic sulfur species emitted into the atmosphere from oceans, is an important participant in the global sulfur cycle (Andreae, 1990; Simo, 2001; Zhang et al., 2019). Approximately 300 Tg of DMS is produced annually mainly by dimethylsulfoniopropionate (DMSP) cleavage through various DMSP lyases (Curson et al., 2011; Johnston et al., 2016), among which 13–37 Tg is transferred into the atmosphere through ocean-atmosphere sulfur flux (Ksionzek et al., 2016). In the air, DMS may contribute to the formation of the cloud condensation nuclei and thus act as a global coolant (Charlson et al., 1987; Lidbury et al., 2016). DMS loss in marine surface waters is mediated by different processes, including photochemical oxidation and biological consumption, with the latter being a major component of the global sink for DMS (Brimblecombe and Shooter, 1986; Kiene and Bates, 1990; Lidbury et al., 2016). Microorganisms can transform DMS into different compounds, such as dimethylsulfoxide (DMSO), methanethiol, sulfate, thiosulfate and tetrathionate (deZwart et al., 1996; Vila-Costa et al., 2006; del Valle et al., 2007; Boden et al., 2010, 2011; Lidbury et al., 2016). In surface seawater, microbial oxidation to DMSO is a major fate of DMS (Lidbury et al., 2016), which accounts for approximately 70% of the total oxidized DMS in the Sargasso Sea (del Valle et al., 2007). DMSO is ubiquitous in aquatic environments, and is likely to function as cryoprotectant, free-radical scavenger or intracellular electrolyte modifier in marine organisms (Lee and De Mora, 1999; Asher et al., 2017; Speeckaert et al., 2018).

The SAR11 clade and the marine *Roseobacter* clade (MRC) are the most abundant heterotrophic bacteria in the ocean surface seawater, and are active participants in marine carbon, nitrogen, sulfur, and phosphorus cycles (Morris et al., 2002; Buchan et al., 2005; Rusch et al., 2007; Chen, 2012; Carini et al., 2015; Sebastián et al., 2016; Tsementzi et al., 2016). Previous studies have shown that trimethylamine monooxygenase (Tmm, EC 1.14.13.148) from both MRC and SAR11 bacteria likely oxidizes DMS to generate DMSO (Chen et al., 2011; Lidbury et al., 2016). It is estimated that ∼20% of the bacteria in the surface ocean contain *tmm* homologs (Chen et al., 2011). Physiological experiments demonstrated that MRC can oxidize DMS to DMSO using Tmm (Lidbury et al., 2016), and it is deduced that SAR11 bacteria may also play a vital role in the conversion of DMS to DMSO in marine environment (Chen et al., 2011; Lidbury et al., 2016). However, the catalytic mechanism underpinning DMS oxidation to DMSO by Tmm remains understudied.

Tmm is a bacterial flavin-containing monooxygenase (FMO), which belongs to the class B flavoprotein monooxygenases (Chen et al., 2011; Paul et al., 2021). FMOs are a widespread class of enzymes that are involved in the metabolism of xenobiotics (Cho et al., 2011). FMOs oxygenate a wide range of substrates, such as nitrogen-containing and sulfur-containing compounds (van Berkel et al., 2006). Tmm is also reported to act on various substrates, including trimethylamine (TMA), dimethylamine (DMA), DMS, indole, and methimazole (Chen et al., 2011). The catalytic process of Tmm to oxidize TMA, indole or methimazole can be divided into two half-reactions: a reductive half-reaction followed by an oxidative half-reaction (Beaty and Ballou, 1981a,b; Cho et al., 2011; Li et al., 2017). In the reductive half-reaction, the cofactor flavin adenine dinucleotide (FAD) is reduced by NADPH. In the oxidative half-reaction, the reduced FAD reacts with an oxygen molecule, generating the C4a-(hydro)peroxyflavin, which is relatively stable *in vitro* (Alfieri et al., 2008). An oxygen atom from the C4a-(hydro)peroxyflavin is transferred to the substrate to complete the oxidation cycle (Alfieri et al., 2008; Orru et al., 2010). However, the detailed structural basis for DMS oxidation is still lacking. Considering the important roles of DMS and DMSO in the global sulfur cycle, the structural basis of DMS oxidation to DMSO by Tmm warrants further investigation.

The SAR11 bacterium *Pelagibacter* sp. HTCC7211 was isolated from the oligotrophic Sargasso Sea (Sun et al., 2011). It has been reported that the recombinant Tmm from strain HTCC7211 (Tmm_7211_) could catalyze the oxidation of TMA to trimethylamine *N*-oxide (TMAO) (Chen et al., 2011). In this study, the Tmm_7211_ gene was synthesized and over-expressed in *Escherichia coli*. The recombinant Tmm_7211_ also exhibits DMS oxidation activity *in vitro*. The crystal structures of Tmm_7211_ and Tmm_7211_ soaked with DMS were solved. The catalytic mechanism of DMSO production through DMS oxidation was proposed by structural analyses and mutational assays.

## Materials and Methods

### Gene Cloning, Point Mutations, and Protein Expression and Purification

The 1335-bp full-length *tmm* gene from *Pelagibacter* sp. HTCC7211 was synthesized by the Beijing Genomics Institute (China). The gene was then subcloned into the pET28a (Novagen, United States) vector with an N-terminal His tag. The point mutations in Tmm_7211_ were introduced using PCR-based method and verified by DNA sequencing. The Tmm_7211_ protein and its mutants were expressed in *E. coli* BL21 (DE3). The cells were cultured at 37°C in Lysogeny Broth medium to an OD_600_ of 0.8–1.0 and then induced at 20°C for 14 h with 0.5 mM isopropyl β-D-1-thiogalactopyranoside (IPTG). The proteins were purified first with Ni^2+^-NTA resin (Qiagen, Germany) and then fractionated on a Superdex-200 column (GE Healthcare, United States). The protein concentration was determined with the Pierce BCA Protein Assay Kit (Thermo Fisher Scientific, United States), and a nine-point calibration curve of bovine serum albumin (BSA) standards was used according to the user guide.

### Gel Filtration Analysis

A Superose 6 column was used for gel filtration analysis, because it possesses a wider fractionation range than the Superdex-200 column. The Superose 6 column was calibrated in the buffer containing 10 mM Tris-HCl (pH 8.0) and 100 mM NaCl using the following standards from GE Healthcare: thyroglobulin (669 kDa), ferritin (440 kDa), aldolase (158 kDa), conalbumin (75 kDa), carbonic anhydrase (29 kDa), ribonuclease A (13.7 kDa), and aprotinin (6.5 kDa). The void volume of Superose 6 column was determined with Blue Dextran 2000 (2,000 kDa).

### Spectrophotometric Analysis

The UV spectra of Tmm_7211_ (0.1 mM protein in the buffer containing 10 mM Tris-HCl (pH 8.0) and 100 mM NaCl) were measured by a V550 UV/VIS spectrophotometer (Jasco, Japan) in a cell with 1.0 cm path length (Response: Medium; Band width: 1.0 nm). The spectra of the mixture of Tmm_7211_ and NADPH were measured immediately after NADPH (0.1 mM) was added into the protein solution.

### High Performance Liquid Chromatography Analysis

The DMSO produced by the enzymatic activity of Tmm_7211_ toward DMS was measured by high performance liquid chromatography (HPLC) (Dionex, America) on a SunFire C_18_ column (Waters, America). The detection wavelength was 210 nm because DMSO exhibited an absorbance maximum at ∼210 nm. The samples were eluted in HPLC buffer (2.5% (v/v) acetonitrile, 0.2% (v/v) phosphoric acid in double-distilled H_2_O) over 20 min at a flow rate of 1 ml/min. The reaction system contained 6 mM DMS (Sigma-Aldrich, America), 1.5 mM NADPH (Sigma-Aldrich, America), 0.15 mM Tmm_7211_, 10 mM Tris-HCl (pH 7.0) and 100 mM NaCl. The reaction was performed at 25°C, pH 7.0 for 3 h, and terminated by adding 10% phosphoric acid. The reaction system was centrifuged at 15,000 g for 15 min, and then the supernatant (20 μl) was injected for HPLC analysis. The control group had the same reaction system except that Tmm_7211_ was not added.

### Enzyme Assays

In the absence of DMS, the consumption of NADPH was less than 3% of that in the presence of DMS, indicating the NADPH-oxidase activity (also known as uncoupling) of Tmm_7211_ is rather weak under the experimental conditions. Because monitoring NADPH oxidation is more sensitive than monitoring DMSO formation, the enzymatic activity of Tmm_7211_ was measured by following the decrease of absorbance at 340 nm (ε_340_ = 6.22 mM^–1^ cm^–1^ for NADPH) (Alfieri et al., 2008). The reaction mixture for detecting the enzymatic activity of Tmm_7211_ contains 1 μM Tmm_7211_, 0.25 mM NADPH, 1 mM DMS, 10 mM Tris-HCl (pH 7.0) and 100 mM NaCl. The reaction mixture without Tmm_7211_ was set as the control. For the measurements of the apparent *K*_*M*_ values of Tmm_7211_, substrate (DMS, TMA, DMA or methimazole) of different concentrations was added into the reaction system containing 1 μM Tmm_7211_ and 0.25 mM NADPH. For the measurements of the apparent *K*_*M*_ values of Tmm_7211_ and its mutants toward NADPH, different concentrations of NADPH were added into the reaction system containing 1.5 μM purified enzyme and 1 mM DMS. The optimal pH and the optimal temperature of Tmm_7211_ were determined using DMS as the substrate. For measurement of the optimal temperature of Tmm_7211_, a buffer containing 10 mM Tri-HCl (pH 8.0) and 100 mM NaCl was pre-incubated at different temperatures for 30 min, and then 1 μM Tmm_7211_, 0.25 mM NADPH and 1 mM DMS were added into the buffer. The mixture was incubated at different temperatures for 6 min before detection of NADPH oxidation at 340 nm using a V550 UV/VIS spectrophotometer (Jasco, Japan). The optimum of pH was examined at 25°C (the optimal temperature for Tmm_7211_ enzymatic activity) using Bis-Tris buffer for pH 6–7, Tris buffer for pH 7–9 and glycine buffer for pH 9–10.

### Crystallization and Data Collection

The purified Tmm_7211_ protein was concentrated to ∼8 mg ml^–1^ in 10 mM Tris-HCl (pH 8.0) and 100 mM NaCl. To obtain crystals of Tmm_7211_, NADPH with a final concentration of 5 mM was added into the protein solution before crystallization. Initial crystallization trials for Tmm_7211_ were performed at 18°C using the sitting drop vapor diffusion method. Diffraction-quality crystals of Tmm_7211_ were obtained in hanging drops containing 0.2 M ammonium citrate tribasic, 0.1 M imidazole (pH 7.0) and 20% (w/v) polyethylene glycol monomethyl ether 2,000 at 18°C after a 3-week incubation. To obtain the crystals of Tmm_7211_ soaked with DMS, Tmm_7211_ crystals were soaked in 20 mM DMS for 5 and 20 min, respectively. X-ray diffraction data were collected on the BL17U1 (Wang et al., 2018) and BL18U1 beamlines at the Shanghai Synchrotron Radiation Facility. The initial diffraction data sets were processed using the HKL3000 program with its default settings (Minor et al., 2006).

### Structure Determination and Refinement

The crystals of Tmm_7211_ and Tmm_7211_ soaked with DMS belong to the *P*2_1_ space group. The crystal structures of Tmm_7211_ and Tmm_7211_ soaked with DMS were determined by molecular replacement using the CCP4 program phaser (Winn et al., 2011) with the crystal structure of a bacterial Tmm (PDB code: 5IPY) as the search model. The refinement of these structures were performed using WinCoot (Emsley et al., 2010) and *Phenix* (Adams et al., 2010). Default parameters in CCP4, WinCoot and *Phenix* were used. All the structure figures were processed using the program PyMOL.^1^

### Circular-Dichroism Spectroscopic Assays

CD spectroscopic assays for Tmm_7211_ and all its mutants were carried out on a J-1,500 Spectrometer (Jasco, Japan) in a 1 mm pathlength cuvette at 25°C. The concentration of the proteins was 8.0 μM in the buffer of 10 mM Tris-HCl (pH 8.0) containing 100 mM NaCl. The buffer without proteins was used for baseline and blank measurements. The spectra were collected from 250 to 200 nm at a scan speed of 500 nm min^–1^ with a band width of 1 nm. Each sample was scanned for three times. The noise level is < 0.05 mdeg.

### Coexistence Analysis of Enzymes Involved in Dimethylsulfide Metabolism

Related protein sequences DddD (*Pseudomonas putida*, WP_062573753.1), DddK (*Candidatus Pelagibacter ubique* HTCC1062, WP_011281678.1), DddP (*Mesorhizobium*, WP_109668646.1), DddQ (*Mesorhizobium loti*, WP_10966 8666.1), DddW (*Ruegeria pomeroyi*, WP_011046214.1), DddL (*Puniceibacterium antarcticum* SM1211, WP_099909581.1), DddY (*Alcaligenes faecalis*, WP_123051132.1), DMSOR (*Rhodobacter capsulatus*, Q52675.2), Tmm (*Pelagibaca abyssi*, APZ51459.1), DdhA (*Sagittula stellata* E-37, EBA07058.1), MddA (*Pseudomonas deceptionensis*, WP_048359798.1), DsoB (*Acinetobacter* sp. 20B, BAA23331.1) and DmoA (*Hyphomicrobium sulfonivorans*, E9JFX9.1) were obtained from National Center for Biotechnology Information (NCBI) database^2^ as seed sequences. For multifunctional strains screening, the seed sequences were used to search against the genomes of isolated strains on the IMG/M metagenomics database (Chen et al., 2019) with parameters of similarity > 40%, *E*-value of < 10^–50^ and coverage > 70% to elevate the accuracy and precision of blast hits. Data processing was performed via scripts compiled in Python code.^3^ The biological networks of related proteins were built via software Cytoscape 3.8.0 (Kohl et al., 2011).

### Accession Numbers

The structures of Tmm_7211_, Tmm_721__1_-5-min and Tmm_721__1_-20-min have been deposited in the Protein Data Bank (PDB) under the accession codes 7D4K, 7D4M, and 7D4N, respectively.

## Results

### Expression and Characterization of Tmm_7211_

**FIGURE 1:**
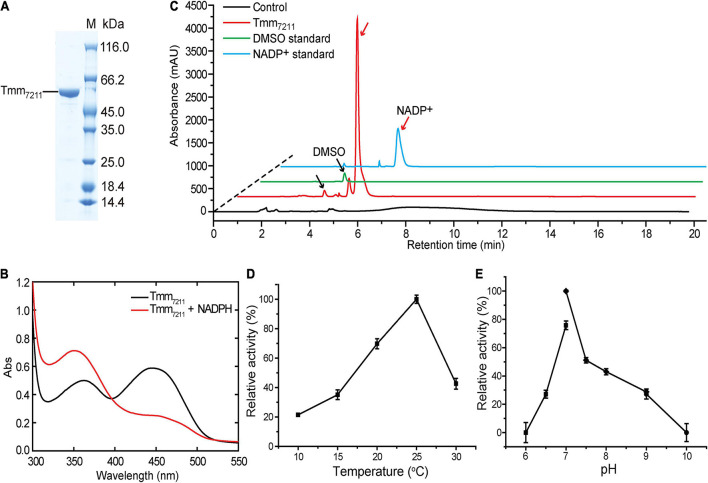
Characterization of Tmm_7211_. **(A)** SDS-PAGE analysis of the purified Tmm_7211_ protein. **(B)** Absorbance spectra of Tmm_7211_. Black line, the absorbance spectrum of the purified Tmm_7211_. Red line, the absorbance spectrum of the purified Tmm_7211_ with the addition of equimolar amount of NADPH (0.1 mM) under aerobic conditions. **(C)** HPLC assay of the enzymatic activity of the recombinant Tmm_7211_ on DMS. The peaks of DMSO and NADP^+^ monitored at 210 nm were indicated with black and red arrows, respectively. The reaction system without Tmm_7211_ was used as the control. The DMSO (1.25 mM) and NADP^+^ (0.4 mM) standards were used as positive controls. **(D)** Effect of temperature on the enzymatic activity of Tmm_7211_. **(E)** Effect of pH on the enzymatic activity of Tmm_7211_. The optimal pH was examined at 25°C using Bis-Tris buffer for pH 6–7, Tris buffer for pH 7–9 and glycine buffer for pH 9–10. The standard errors are from three independent experiments.

The *tmm* gene of *Pelagibacter* sp. HTCC7211 contains 1335 nucleotides and encodes a protein of 444 amino acid residues, with a calculated molecular mass of 52 kDa. Tmm_7211_ shares ∼53% amino acid sequence identity with *Rn*Tmm, a previously reported Tmm homolog from an MRC strain *Roseovarius nubinhibens* ISM (Li et al., 2017). Full-length *tmm* of strain HTCC7211 was synthesized and was expressed in *E. coli* BL21 (DE3) cells, and the recombinant Tmm_7211_ was purified (Figure 1A) and characterized. The purified Tmm_7211_ is yellow, suggesting that FAD has been already bound in the recombinant Tmm_7211_ during protein expression in *E. coli*, which is further supported by spectroscopic analysis. The purified Tmm_7211_ exhibited the typical absorbance maxima (around 372 and 442 nm) of fully oxidized FMOs (Figure 1B; Alfieri et al., 2008; Orru et al., 2010). Addition of equimolar amount of NADPH should lead to the formation of the enzyme-(hydro)peroxyflavin-NADP^+^ complex exhibiting a typical absorbance maximum at around 360 nm (Alfieri et al., 2008; Orru et al., 2010). However, the absorption spectrum showed that the absorbance maximum of Tmm_7211_ with the addition of equimolar amount of NADPH was around 350 nm (Figure 1B). Because NADPH absorbs at 340 nm (Alfieri et al., 2008), this spectrum probably reflected a mixture of the enzyme-(hydro)peroxyflavin-NADP^+^ complex and some residual NADPH, which may due to some inactive enzymes in the purified Tmm_7211_ solution. Incubation of recombinant Tmm_7211_ with DMS and NADPH yielded DMSO and NADP^+^ (Figure 1C), demonstrating that Tmm_7211_ has DMS oxidation activity *in vitro*. The optimal temperature for Tmm_7211_ enzymatic activity toward DMS was ∼25°C (Figure 1D), and the optimal pH was 7.0 (Figure 1E). The optimal temperature of Tmm_7211_ is lower than that of *Rn*Tmm toward TMA (30°C) (Li et al., 2017). Furthermore, Tmm_7211_ only retained ∼40% of its highest enzymatic activity at 30°C, whereas *Rn*Tmm still retained ∼70% of its highest enzymatic activity at 40°C (Li et al., 2017), suggesting that Tmm_7211_ is more sensitive to high temperature than *Rn*Tmm.

The substrate specificity of Tmm_7211_ was also analyzed. Tmm_7211_ can oxidize DMS, TMA, DMA, and methimazole, with TMA showing the highest affinity (Table 1). In general, the apparent *K*_*M*_ values of Tmm_7211_ to different substrates are slightly higher than those of *Rn*Tmm, and the *k*_*cat*_ values of Tmm_7211_ are lower (Table 1; Li et al., 2017), indicating that the enzymatic activity of Tmm_7211_ is lower than that of *Rn*Tmm *in vitro*.

**TABLE 1:** Kinetic parameters for Tmm_7211_ and *Rn*Tmm.

**Enzyme**	**Substrate**	**Apparent *K*_*M*_ (μM)**	***k*_*cat*_ (min^–1^)**	***k*_*cat*_/*K*_*M*_ (min^–1^ mM^–1^)**	**References**
Tmm_7211_					
	DMS	250.5 ± 23.0	4.5 ± 0.3	18.0	This study
	TMA	139.0 ± 10.7	22.4 ± 0.8	161.2	This study
	DMA	181.4 ± 23.7	17.9 ± 1.7	98.7	This study
	Methimazole	116.2 ± 13.8	5.2 ± 0.3	44.8	This study
*Rn*Tmm					
	TMA	110.5 ± 14.5	31.8 ± 2.4	287.8	Li et al., 2017
	DMA	164.9 ± 36.5	10.2 ± 1.2	61.9	Li et al., 2017
	Methimazole	123.3 ± 44.6	13.2 ± 1.8	107.1	Li et al., 2017

### Overall Structure of Tmm_7211_

**FIGURE 2:**
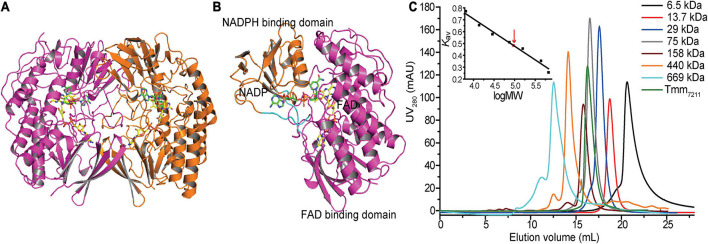
Overall structure of Tmm_7211_. **(A)** Two monomers of Tmm_7211_ arranged as a dimer in an asymmetric unit. The monomers are colored in magenta and orange, respectively. **(B)** The overall structure of Tmm_7211_ monomer. Tmm_7211_ contains an NADPH binding domain (colored in orange) and a FAD binding domain (colored in magenta) connected through two hinge regions (colored in cyan). The NADP^+^ molecule and the FAD molecule are shown as sticks colored in green and yellow, respectively. **(C)** Gel filtration analysis of Tmm_7211_. Inset, semilog plot of the molecular mass of all standards used vs. their *K*_*av*_ values (black squares). The red arrow indicates the position of the *K*_*av*_ value of Tmm_7211_ (0.48) interpolated in the regression line. Tmm_7211_ monomer has a calculated molecular mass of 52 kDa. The apparent molecular mass of Tmm_7211_ is 95 kDa, indicating that Tmm_7211_ is a dimer in solution.

To gain insight into the putative active site of Tmm_7211_, we solved the crystal structure of Tmm_721__1_ to 1.8 Å (Table 2). The crystals of Tmm_7211_ belong to the *P*2_1_ space group, with two molecules arranged as a dimer in an asymmetric unit (Figures 2A,B). Gel filtration analysis (Figure 2C) indicated that Tmm_7211_ functions as a dimer in solution, which is supported by the result of the PISA server prediction.^4^ After structural refinement, the NADP^+^ and FAD molecules can be clearly observed in the structure (Figure 2A). The overall structure of Tmm_7211_ is similar to those of other reported bacterial FMOs (Alfieri et al., 2008; Cho et al., 2011; Li et al., 2017), with the root mean square deviations (RMSDs) between Tmm_7211_ and other bacterial FMOs of no more than 0.6 Å. Tmm_7211_ also comprises an NADPH binding domain and an FAD binding domain (Figure 2B). These two domains are connected through two hinge regions (Ser163–Pro168 and Cys268–Leu272) (Figure 2B).

**TABLE 2:** Crystallographic data collection and refinement of Tmm_7211_.

**Parameters**	**Tmm_7211_**	**Tmm_721__1_-5-min**	**Tmm_721__1_-20-min**
**Diffraction data**			
Space group	*P*2_1_	*P*2_1_	*P*2_1_
Unit cell			
a, b, c (Å)	69.4, 82.1, 97.9	69.0, 81.8, 97.9	68.7, 81.7, 97.6
α, β, γ (°)	90.0, 98.1, 90.0	90.0, 98.1, 90.0	90.0, 98.5, 90.0
Resolution range (Å)	50.0–1.8 (1.83–1.80)^*^	50.0–1.8 (1.86–1.80)	50.0–2.0 (2.07–2.00)
Redundancy	3.3 (3.3)	6.4 (6.9)	6.6 (6.3)
Completeness (%)	98.1 (98.6)	97.7 (98.6)	100.0 (100.0)
*R*_*merge*_ ^**^	0.1 (0.4)	0.1 (0.4)	0.1 (0.6)
*I*/σ*I*	18.0 (1.8)	49.8 (11.9)	26.6 (3.7)
**Refinement statistics**			
R-factor	0.22	0.16	0.16
Free R-factor	0.25	0.18	0.20
**RMSD from ideal geometry**			
Bond lengths (Å)	0.006	0.006	0.006
Bond angles (°)	1.1	1.2	1.1
**Ramachandran plot (%)**			
Favored	93.8	94.7	94.7
Allowed	6.0	5.1	5.1
Outliers	0.2	0.2	0.2
B-factors (Å^2^)			
Protein	29.7	23.7	31.5
NADP^+^	25.1	21.8	28.7
FAD	23.2	18.5	27.3
Water	38.5	33.5	37.4
All atoms	30.6	24.9	32.0

*Numbers in parentheses refer to data in the highest-resolution shell.

** R_*merge*_ = Σ_*hkl*_Σ_*i*_| I(hkl)_*i*_—< I(hkl) > | /Σ_*hkl*_Σ_*i*_I(hkl)_*i*_, where I is the observed intensity, < I(hkl) > represents the average intensity, and I(hkl)_*i*_ represents the observed intensity of each unique reflection.

To obtain the crystal structure of Tmm_7211_ in complex with DMS, we first tried to co-crystalize Tmm_7211_ with DMS. However, this failed, probably due to the volatile nature of DMS that has a low boiling point (∼37°C) in the crystallization buffer. Next, we tried the soaking method and solved two crystal structures of Tmm_7211_ soaked with DMS for different soaking time (Table 2). For briefness, the crystal structures of Tmm_7211_ soaked with DMS for 5 min and for 20 min were termed as Tmm_721__1_-5-min and Tmm_721__1_-20-min, respectively. The overall structures of Tmm_7211_ soaked with DMS are similar to that of Tmm_7211_, with the RMSD between Tmm_7211_ and Tmm_721__1_-5-min of 0.1 Å, and the RMSD between Tmm_7211_ and Tmm_721__1_-20-min of 0.2 Å.

### Residues Involved in Binding NADP^+^ and Flavin Adenine Dinucleotide

**FIGURE 3:**
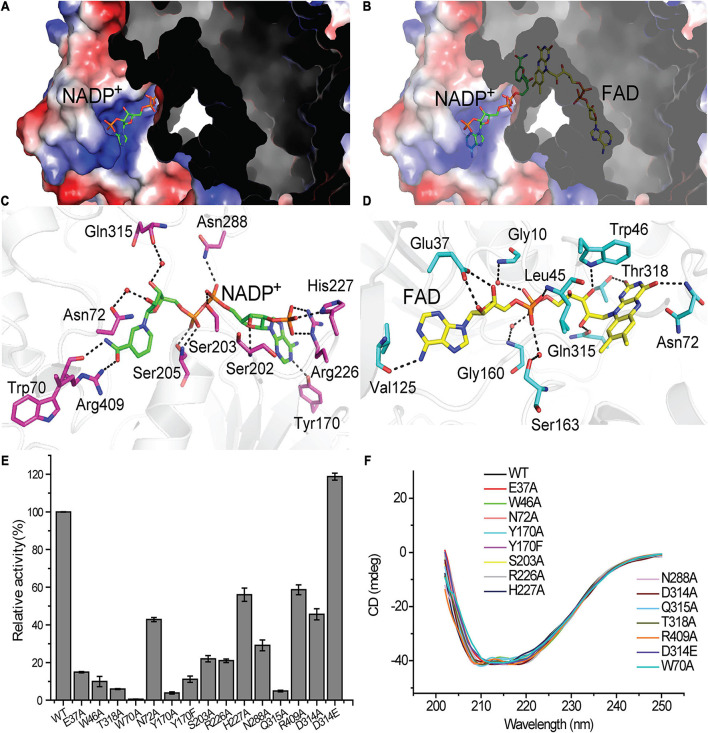
The binding of the NADP^+^ molecule and the FAD molecule in Tmm_7211_. The NADP^+^ molecule and the FAD molecule are shown as sticks colored in green and yellow, respectively. **(A)** Electrostatic surface of Tmm_7211_. The NADP^+^ molecule is partially visible through the surface. **(B)** Electrostatic surface of Tmm_7211_ after the transparency of surface was set to 40%. **(C)** Interactions between NADP^+^ and Tmm_7211_ residues. **(D)** Interactions between FAD and Tmm_7211_ residues. Water molecules are shown in red dots. The possible hydrogen bonds are represented by dashed lines. **(E)** The enzymatic activities of WT Tmm_7211_ and its mutants. The activity of WT Tmm_7211_ is taken as 100%. The standard errors are from three independent experiments. **(F)** CD spectra of WT Tmm_7211_ and its mutants.

From the surface view of Tmm_7211_, we can only observe part of the NADP^+^ molecule and the FAD molecule was not visible (Figure 3A). The nicotinamide ring of NADP^+^ is located inside Tmm_7211_, and the entire FAD molecule is deeply bound in the protein (Figures 3A,B). The binding of NADP^+^ and FAD mainly depends on hydrogen bonds formed between Tmm_7211_ residues and them (Figures 3C,D). For NADP^+^ binding, residues Trp70 and Arg409 form hydrogen bonds with the nicotinamide ring, Asn72 and Gln315 interact with the ribose ring via water-mediated hydrogen-bonds, and Tyr170, Ser202, Ser203, Ser205, Arg226, His227, and Asn288 interact with the other parts of NADP^+^ (Figure 3C). For FAD binding, residues Asn72 and Thr318 interact with the isoalloxazine ring, Glu37 forms hydrogen bonds with the ribose ring, Val125 forms a hydrogen bond with the adenine moiety, and Gly10, Leu45, Trp46, Gly160, Ser163, and Gln315 interact with the other parts of FAD (Figure 3D).

To confirm the importance of Tmm_7211_ residues involved in binding NADP^+^ and FAD, we generated site-directed mutations to the related residues and quantified the enzymatic activities of the mutants. All mutants had significantly decreased activity (Figure 3E), suggesting that these residues play important roles for the correct binding of NADP^+^ or FAD. Moreover, mutants Asn72Ala, Ser203Ala, Arg226Ala, His227Ala, Asn288Ala, and Arg409Ala all exhibited higher apparent *K*_*M*_ values toward NADPH than wild type (WT) Tmm_7211_ (Table 3), further supporting their roles in binding NADP^+^/NADPH. CD spectroscopy analysis showed that the secondary structures of the mutants exhibited little deviation from that of WT Tmm_7211_ (Figure 3F), indicating that the decreases in the enzymatic activities of the mutants are caused by residue replacement rather than structural changes.

**TABLE 3:** Kinetic parameters for Tmm_7211_ and its mutants toward NADPH.

**Enzyme**	**apparent *K*_*M*_ (μM)**	***k*_*cat*_ (min^–1^)**
Wild type	12.8 ± 0.4	4.5 ± 0.3
Asn72Ala	34.1 ± 1.0	1.9 ± 0.2
Ser203Ala	134.8 ± 11.1	1.0 ± 0.1
Arg226Ala	101.8 ± 8.6	0.9 ± 0.1
His227Ala	41.2 ± 2.3	2.5 ± 0.2
Asn288Ala	61.1 ± 4.0	1.3 ± 0.1
Arg409Ala	44.9 ± 5.6	2.7 ± 0.1
Asp314Ala	32.0 ± 0.6	2.1 ± 0.1
Asp314Glu	9.7 ± 0.5	5.2 ± 0.2

### Conformational Change of NADP^+^ During Soaking Dimethylsulfide

To elucidate the catalytic mechanism of Tmm_7211_ for DMS oxidation, it is important to ascertain the location of DMS. Despite the two structures of Tmm_7211_ soaked with DMS were solved, the explicit electron density of DMS in the structures could not be identified. Previous structural analyses demonstrated that the substrate of bacterial FMOs with a ring structure, such as indole and methimazole, is located in the position of the nicotinamide ring of NADP^+^, forming stacking interactions with the isoalloxazine ring of FAD (Eswaramoorthy et al., 2006; Cho et al., 2011; Li et al., 2017). There is no direct interaction between the residues of bacterial FMOs and the substrates (Eswaramoorthy et al., 2006; Cho et al., 2011; Li et al., 2017). For substrates with no ring structure, such as DMS and TMA, there may be no effective interactions to stabilize their conformations, which may be the reason why we could not find the DMS molecule in the structures of Tmm_7211_ soaked with DMS.

**FIGURE 4:**
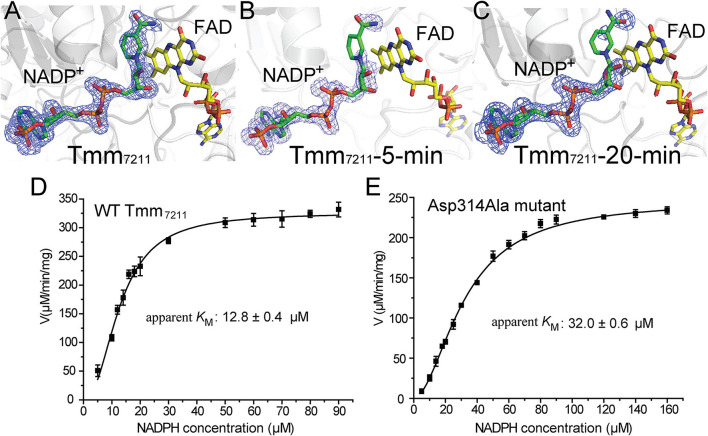
Analysis of the conformational change of NADP^+^. **(A)** Structure of Tmm_7211_. **(B)** Structure of Tmm_721__1_-5-min. **(C)** Structure of Tmm_721__1_-20-min. The NADP^+^ molecule and the FAD molecule are shown as sticks colored in green and yellow, respectively. The *F*_*o*_-*F*_*c*_ densities for NADP^+^ are contoured in blue meshes at 3.0σ. **(D)** Kinetic analysis of WT Tmm_7211_ toward NADPH. **(E)** Kinetic analysis of the mutant Asp314Ala toward NADPH.

By comparing the structures of Tmm_7211_, Tmm_721__1_-5-min and Tmm_721__1_-20-min, we noticed that with the extension of soaking time, the electron densities of the nicotinamide ring and the ribose ring of NADP^+^ become increasingly weaker (Figures 4A–C), indicating that the nicotinamide ring and the ribose ring become flexible during soaking. Combined with previous studies that the indole and methimazole molecules located in the binding site of NADP^+^ (Eswaramoorthy et al., 2006; Cho et al., 2011; Li et al., 2017), this result suggests that DMS may also be bound in the position of the nicotinamide ring of NADP^+^, and that the entry of DMS may repel NADP^+^, leading to a conformational change of the nicotinamide ring and the ribose ring.

The conformational change of NADP^+^ was also observed in *Rn*Tmm when soaked with TMA, and this conformational change was shown to be important for TMA oxidation (Li et al., 2017). After conformational change, the ribose ring of NADP^+^ in *Rn*Tmm forms a hydrogen bond with Asp317, shutting off the substrate entrance to promote a protected micro-environment for catalysis (Li et al., 2017). Because the electron densities of the ribose ring in Tmm_7211_-5-min and Tmm_7211_-20-min are rather poor (Figures 4B,C), we could not ascertain whether the ribose ring can form a hydrogen bond with Asp314 of Tmm_7211_, the equivalent residue to Asp317 of *Rn*Tmm. To further probe this, we generated mutants Asp314Ala and Asp314Glu, and measured their enzymatic activities. The enzymatic activity and the apparent *K*_*M*_ of Tmm_7211_ toward NADPH are only slightly affected by Asp314Glu mutation (Figure 3E and Table 3), probably due to the similar properties of aspartic acid and glutamic acid. However, although the residue Asp314 is far away from the catalytic center of Tmm_7211_, the mutation of Asp314 to alanine decreased the activity of Tmm_7211_ significantly (Figure 3E), suggesting that Asp314 is involved in the catalytic reaction of Tmm_7211_. The CD spectrum of the mutant Asp314Ala was indistinguishable from that of WT Tmm_7211_, suggesting that the enzymatic activity loss in the mutant is caused by residue replacement rather than structural alteration of the enzyme (Figure 3F). In addition, the Asp314Ala mutation increased the apparent *K*_*M*_ of Tmm_7211_ toward NADPH (Figures 4D,E), suggesting that this residue participates in binding NADP^+^/NADPH. Because structural analysis shows that the residue Asp314 is too far away to participate in NADP^+^ binding before DMS enters the catalytic center (Figure 3C), this result suggests that, after DMS enters the catalytic center, NADP^+^ in Tmm_7211_ likely undergoes a conformational change and forms a new hydrogen bond with Asp314, which is important for the catalysis of DMS oxidation.

## Discussion

**FIGURE 5:**
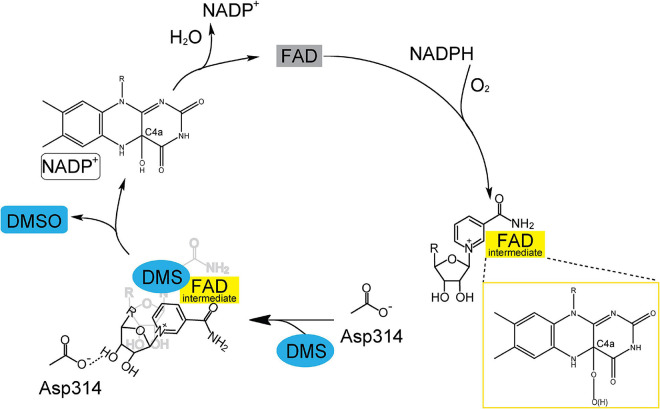
A proposed catalytic cycle of Tmm_7211_ oxidizing DMS to generate DMSO. In the reductive half-reaction, FAD is reduced by NADPH. In the oxidative half-reaction, the reduced FAD reacts with O_2_, and a C4a-(hydro)peroxyflavin (FAD intermediate) is formed. The nicotinamide ring of NADP^+^ protects the FAD intermediate from solvent attack. When DMS enters the catalytic pocket, NADP^+^ generates a conformational change to form a hydrogen bond with Asp314, shutting off the substrate entrance and exposing the FAD intermediate to DMS. After the reaction, DMSO, NADP^+^ and a water molecule are released and the oxidized FAD is regenerated.

DMS and DMSO are widespread in marine environment, and the oxidation of DMS to DMSO is an important biogeochemical reaction. Tmm_7211_ is a bacterial FMO which is able to catalyze the conversion of DMS to DMSO. Based on our results and previous studies of bacterial FMOs (Alfieri et al., 2008; Cho et al., 2011; Li et al., 2017), the structural basis of Tmm_7211_ for DMS oxidation to DMSO is proposed (Figure 5). The catalytic cycle of Tmm_7211_ consists of a reductive half-reaction and an oxidative half-reaction, similar to other bacterial FMOs (Alfieri et al., 2008; Cho et al., 2011; Li et al., 2017). In the reductive half-reaction, FAD is reduced by NADPH. In the oxidative half-reaction, the reduced FAD reacts with an oxygen molecule and the (hydro)peroxyflavin intermediate forms (Figures 1B, 5), which is a common trait for FMOs (Krueger and Williams, 2005; Eswaramoorthy et al., 2006; Alfieri et al., 2008). The nicotinamide ring of NADP^+^ buried in Tmm_7211_ protects the C4a-(hydro)peroxyflavin from solvent attack (Beaty and Ballou, 1981b; Li et al., 2017). The (hydro)peroxyflavin intermediate is stable in cells and behaves like a “cocked gun,” awaiting a suitable substrate (Alfieri et al., 2008; Lidbury et al., 2016). After entering the catalytic pocket, DMS may occupy the position of the nicotinamide ring of NADP^+^ and likely makes NADP^+^ generate a conformational change, leading to two consequences: (1), NADP^+^ forms a hydrogen bond with Asp314, shutting off the substrate entrance and creating a protected micro-environment for catalysis; (2), the nicotinamide ring of NADP^+^ no longer protects the C4a-(hydro)peroxyflavin, exposing the active C4a-(hydro)peroxyflavin to DMS to complete the oxidation cycle (Figure 5). After the reaction, DMSO, NADP^+^ and a water molecule are released and the oxidized FAD is regenerated, enabling Tmm_7211_ to get ready for the next catalytic cycle.

**FIGURE 6:**
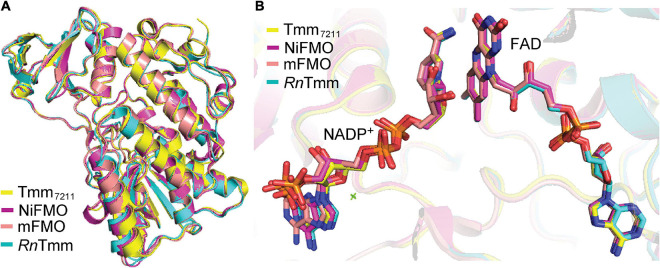
Structural comparisons between Tmm_7211_ and three other reported bacterial FMOs. **(A)** Superimposition of the structures of Tmm_7211_, NiFMO (PDB code: 6HNS), mFMO (PDB code: 2VQ7) and *Rn*Tmm (PDB code: 5IPY). Structures of Tmm_7211_, NiFMO, mFMO and *Rn*Tmm are colored in yellow, purple, salmon and cyan, respectively. **(B)** The locations of the NADP^+^ and FAD in the four structures. The NADP^+^ and FAD molecules are shown as sticks.

To elucidate the catalytic mechanism of Tmm_7211_ to oxidize DMS, it is important to obtain structures of Tmm_7211_ and Tmm_7211_ in complex with DMS. Here, although we solved Tmm_7211_ structures in three states, all our attempts to obtain the structure of Tmm_7211_ in complex with DMS failed. As such, we propose the structural basis for DMS oxidation to DMSO by Tmm_7211_ based on our structural and mutational analyses, and the previous studies of bacterial FMOs (Alfieri et al., 2008; Cho et al., 2011; Li et al., 2017). Tmm_7211_ shares ∼53% sequence identity with three other reported bacterial FMOs from *Nitrincola lacisaponensis* (NiFMO) (Loncar et al., 2019), *Methylophaga* sp. strain SK1 (mFMO) (Alfieri et al., 2008) and *R. nubinhibens* ISM (*Rn*Tmm) (Li et al., 2017). The overall structure as well as the locations of NADP^+^ and FAD of Tmm_7211_ are similar to those of mFMO (PDB code: 2VQ7), *Rn*Tmm (PDB code: 5IPY) and NiFMO (PDB code: 6HNS) (Figure 6), with the RMSDs between Tmm_7211_ and mFMO, *Rn*Tmm and NiFMO of 0.5 Å, 0.6 Å and 0.5 Å, respectively. This suggests that Tmm_7211_ may adopt a similar catalytic mechanism to oxidize DMS as these bacterial FMOs. Indeed, the catalytic mechanism of Tmm_7211_ oxidizing DMS we proposed is similar to that of *Rn*Tmm oxidizing TMA (Li et al., 2017), which includes a reductive half-reaction and an oxidative half-reaction. Asp317 of *Rn*Tmm was reported to form a hydrogen bond with the ribose ring of NADP^+^ after its conformational change (Li et al., 2017). Here, our structural and biochemical results indicate that Asp314 of Tmm_7211_ also likely forms a hydrogen bond with NADP^+^ after its conformational change, which is important for the catalysis of DMS oxidation. Moreover, sequence analysis showed that the residue Asp314 and most residues involved in binding NADP^+^ and FAD in Tmm_7211_ are highly conserved in the Tmm sequences in both MRC and SAR11 bacteria (Li et al., 2017), suggesting that these residues are important residues in bacterial Tmms and that MRC and SAR11 bacteria containing Tmm may adopt a similar mechanism in oxidizing both DMS and TMA. Despite these analyses, further efforts, especially attempts to obtain the Tmm-DMS complex structure, are needed to provide more details of the catalytic mechanism of Tmm to oxidize DMS.

The volatile DMS is predominately produced from microbial degradation of DMSP through various DMSP dethiomethylases (colloquial “DMSP lyases,” EC 4.4.1.3) and the DMSP CoA-transferase/lyase DddD (EC 2.3.1.x) (Curson et al., 2011; Acolombri et al., 2014; Johnston et al., 2016). Despite this, DMS can also be generated from DMSO reduction catalyzed by the DMSO reductase DMSOR (EC 1.8.5.3) (Bray et al., 2001), and from methanethiol (MeSH) via the methyltransferase MddA (EC 2.1.1.334, methanethiol *S*-methyltransferase) (Carrion et al., 2017; Boden and Hutt, 2019). In addition to Tmm, three other enzymes, namely DMS dehydrogenase DdhABC (EC 1.8.2.4, DMS:cytochrome *c* reductase) (McDevitt et al., 2002; Boden and Hutt, 2019), assimilatory DMS *S*-monooxygenase DsoABCDEF (EC 1.14.13.245) (Horinouchi et al., 1997; Boden and Hutt, 2019) and dissimilatory DMS monooxygenase DmoAB (EC 1.14.13.131) (Boden et al., 2011; Boden and Hutt, 2019), also participate in the bacterial consumption of DMS. DdhABC and DsoABCDEF convert DMS to DMSO, while DmoAB converts DMS to MeSH (Horinouchi et al., 1997; McDevitt et al., 2002; Boden et al., 2011). To investigate the prevalence and the coexistence of the enzymes involved in DMS metabolism, we searched homologs of DMS metabolism related enzymes using the IMG/M database (Chen et al., 2019). In total, 3,182 non-redundant bacterial genomes were filtered out to possess at least one type of enzymes related to DMS metabolism, among which 415 contain more than one types of DMS related genes. All these enzyme combinations yielded 22 different one-to-one enzyme configuration modes (Supplementary Figure 1). The relatively frequent co-occurrence between Tmm and DMSP dethiomethylases suggests that some bacteria may channel DMS generated from DMSP cleavage to DMS oxidation to DMSO.

Both previous metagenomic analysis (Chen et al., 2011) and the coexistence analysis presented here suggest a high potential of the oxidation of DMS to DMSO via Tmm catalysis. It was also reported that the oxidation of DMS in MRC is methylated amine-dependent (Lidbury et al., 2016). However, the wide distribution of methylated amines in marine environments (Wang and Lee, 1990; Chen et al., 2011) suggests that Tmm may be functional in DMS oxidation under physiological conditions. Considering that the oxidation to DMSO is a major fate of DMS in surface seawater and the ubiquity of DMSO in marine environments (Lidbury et al., 2016), there should exist active microbiol processes to consume DMSO. The microorganisms and the metabolic pathways involved in DMSO metabolisms warrant further investigation.

## Conclusion

The oxidation of oceanic DMS to DMSO is an important step in the global sulfur cycle, which can be catalyzed by Tmm (Lidbury et al., 2016). Tmm is present in ∼20% of the bacteria in the surface ocean, and is particularly common in the cosmopolitan marine heterotrophs such as MRC and SAR11 bacteria (Chen et al., 2011). In this study, the recombinant Tmm_7211_ protein from the SAR11 bacterium *Pelagibacter* sp. HTCC7211 was purified and characterized. The crystal structures of Tmm_7211_ and Tmm_7211_ soaked with DMS were also solved. Based on structural analysis and mutational assays, the catalytic mechanism for Tmm_7211_ oxidizing DMS was proposed, which may be widely adopted by MRC and SAR11 bacteria. This study offers a better understanding of how marine bacteria oxidize DMS to generate DMSO.

## Data Availability Statement

The datasets presented in this study can be found in online repositories. The names of the repository/repositories and accession number(s) can be found in the article/Supplementary Material.

## Author Contributions

C-YL and Y-ZZ designed the research. X-LC and H-HF directed the research. C-YL, X-JW, NZ, and Z-JT performed the experiments. PW, W-PZ, and YC helped in data analysis. C-YL and X-LC wrote the manuscript. YC edited the manuscript. All authors contributed to the article and approved the submitted version.

## Conflict of Interest

The authors declare that the research was conducted in the absence of any commercial or financial relationships that could be construed as a potential conflict of interest.

## Publisher’s Note

All claims expressed in this article are solely those of the authors and do not necessarily represent those of their affiliated organizations, or those of the publisher, the editors and the reviewers. Any product that may be evaluated in this article, or claim that may be made by its manufacturer, is not guaranteed or endorsed by the publisher.

## Footnotes

1
http://www.pymol.org/

2
https://www.ncbi.nlm.nih.gov/

3
https://www.python.org/

4
http://www.ebi.ac.uk/pdbe/prot_int/pistart.html
