# Supplementary material for: Structural and Mechanistic Insights Into Dimethylsulfoxide Formation Through Dimethylsulfide Oxidation
Source: Front Microbiol. 2021 Sep 24;12:735793. doi: 10.3389/fmicb.2021.735793 (PMC8498191; doi:10.3389/fmicb.2021.735793)
Supplement: Supplementary file 1 [file Data_Sheet_1.DOCX]

**Supplementary Figure** **1** The biological networks of enzyme-enzyme coexistence modes in bacterial strains involved in DMS metabolism. Different types of enzymes are presented as nodes with different colors. The size of nodes and the thickness of edges represent the frequencies of coexistence modes.
