# Supplementary figures and images for: Structural and Mechanistic Insights Into Dimethylsulfoxide Formation Through Dimethylsulfide Oxidation
Source: Front Microbiol. 2021 Sep 24;12:735793. doi: 10.3389/fmicb.2021.735793 (PMC8498191; doi:10.3389/fmicb.2021.735793)

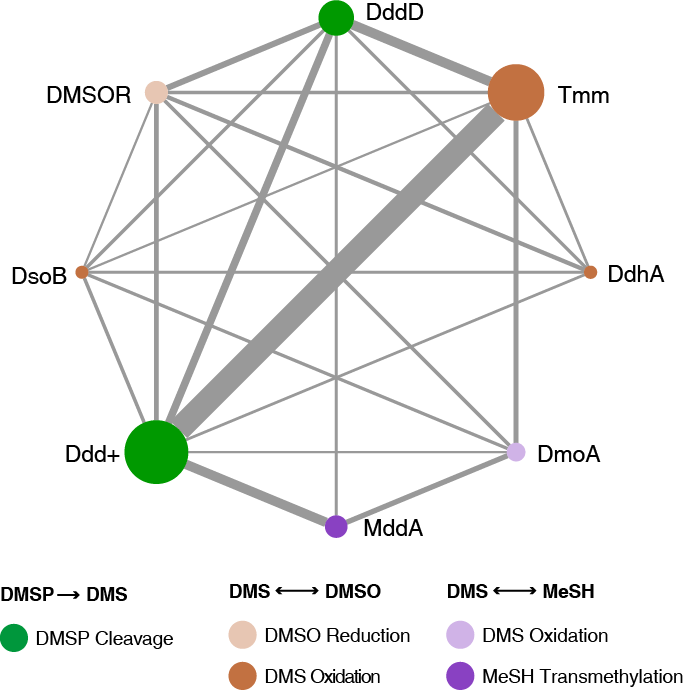

Supplement: Supplementary file 2 [file Image_1.TIF]
